# Radiation-Induced Changes in Serum Lipidome of Head and Neck Cancer Patients

**DOI:** 10.3390/ijms15046609

**Published:** 2014-04-17

**Authors:** Karol Jelonek, Monika Pietrowska, Malgorzata Ros, Adam Zagdanski, Agnieszka Suchwalko, Joanna Polanska, Michal Marczyk, Tomasz Rutkowski, Krzysztof Skladowski, Malcolm R. Clench, Piotr Widlak

**Affiliations:** 1 Center for Translational Research and Molecular Biology of Cancer, Maria Sklodowska-Curie Memorial Cancer Center and Institute of Oncology, Gliwice Branch, Wybrzeze Armii Krajowej 15, 44-100 Gliwice, Poland; E-Mails: m_pietrowska@io.gliwice.pl (M.P.); ros.malgorzata@gmail.com (M.R.); tomr22@tlen.pl (T.R.); skladowski@io.gliwice.pl (K.S.); widlak@io.gliwice.pl (P.W.); 2 Polish-Japanese Institute of Information Technologies, Koszykowa 86, 02-008 Warszawa, Poland; 3 Institute of Mathematics and Computer Science, Wroclaw University of Technology, Janiszewskiego 14a, 50-370 Wroclaw, Poland; E-Mail: Adam.Zagdanski@pwr.wroc.pl; 4 Institute of Biomedical Engineering and Instrumentation, Wroclaw University of Technology, Wybrzeze Wyspianskiego 27, 50-370 Wroclaw, Poland; E-Mail: agnieszka.suchwalko@pwr.wroc.pl; 5 MedicWave AB, Kristian IV:s vag 3, 302 50 Halmstad, Sweden; 6 Faculty of Automatic Control, Electronics and Computer Science, Silesian University of Technology, Akademicka 16, 44-100 Gliwice, Poland; E-Mails: Joanna.Polanska@polsl.pl (J.P.); Michal.Marczyk@polsl.pl (M.M.); 7 Biomedical Research Centre, Sheffield Hallam University, Sheffield S1 1WB, UK; E-Mail: M.R.Clench@shu.ac.uk

**Keywords:** dose-volume effect, intensity-modulated radiation therapy, mass spectrometry, radiation toxicity, serum lipidome

## Abstract

Cancer radiotherapy (RT) induces response of the whole patient’s body that could be detected at the blood level. We aimed to identify changes induced in serum lipidome during RT and characterize their association with doses and volumes of irradiated tissue. Sixty-six patients treated with conformal RT because of head and neck cancer were enrolled in the study. Blood samples were collected before, during and about one month after the end of RT. Lipid extracts were analyzed using MALDI-oa-ToF mass spectrometry in positive ionization mode. The major changes were observed when pre-treatment and within-treatment samples were compared. Levels of several identified phosphatidylcholines, including (PC34), (PC36) and (PC38) variants, and lysophosphatidylcholines, including (LPC16) and (LPC18) variants, were first significantly decreased and then increased in post-treatment samples. Intensities of changes were correlated with doses of radiation received by patients. Of note, such correlations were more frequent when low-to-medium doses of radiation delivered during conformal RT to large volumes of normal tissues were analyzed. Additionally, some radiation-induced changes in serum lipidome were associated with toxicity of the treatment. Obtained results indicated the involvement of choline-related signaling and potential biological importance of exposure to clinically low/medium doses of radiation in patient’s body response to radiation.

## 1. Introduction

Metabolomics, an emerging field of the “omics” sciences, has a capacity to deliver essential information about small biomolecules (<1 kDa) that are end-products of all cellular processes. Lipidomics, which deals with dynamic changes of cellular lipids and their derivatives, is one of the most complex areas of metabolomics [1]. More than 500 different lipid species was reported to be present in human plasma specimens [2]. The most abundant category of lipids are glycerophospholipids (phospholipids; PLs). PLs are both key components of biological membranes and important players in different cellular mechanisms [3,4]. Derivatives of PLs are important signaling molecules involved in regulation of proliferation and apoptosis [5,6]. Of note, metabolism of phosphatidylcholines (PCs) and other PLs is significantly disturbed in cancer cells, hence elevated serum levels of their precursors (e.g., choline) and derivatives (e.g., lysophosphatidylcholines, LPCs) are promising cancer markers [7]. Changes in level of choline-containing lipids were observed in malignant tumors during anti-cancer therapy [8]. Metabolism and blood levels of PLs changed also after exposure to ionizing radiation [9,10]. Although such effects have only been studied in animal models until now, they indicated applicability of serum phospholipid profiles in assessment of radiation exposure.

Radiotherapy (RT), either alone or in combination with chemotherapy, is an effective treatment of different types of cancer allowing preservation of structure and function of a target organ. The effects of ionizing radiation concerns damage induced not only in cancer cells, but also in adjacent normal tissue. Conformal methods of radiotherapy, like intensity-modulated radiation therapy (IMRT), were developed to allow precise delivery of high radiation doses to a tumor volume, minimizing the dose delivered to surrounding normal tissues [11]. This technique is being used most extensively in treatment of tumors located near critical structures, such as head and neck cancers [12]. IMRT is accomplished by application of many non-coplanar radiation fields that markedly extends the volume of normal tissues being exposed to low doses of radiation, for which biological relevance is not clear at the moment [13]. Radiation-induced damage of normal tissues could lead to acute and/or late injury reactions, which in extreme cases might significantly affect patient’s comfort and effectiveness of the treatment. For this reason planning and monitoring of radiotherapy would be greatly facilitated if molecular markers of individual response to radiation were available in the clinical practice. In addition, molecular markers of exposure to ionizing radiation would have a great applicability in the epidemiology field and for exposure assessment after radiation accidents [14].

Local irradiation during cancer radiotherapy induces patient’s whole body response that could be detected at the level of blood components. Markers of human exposure to ionizing radiation have been searched in blood cells using different genetic and genomics approaches [15–18]. Mass spectrometry-based proteomics approaches have been also explored, which allowed identifying of radiotherapy-related changes in serum proteome of cancer patients [19,20]. More recently, it has been shown that IMRT-induced changes in the low-molecular-weight fraction of serum proteome of head and neck cancer patients were affected by clinically irrelevant doses of radiation delivered to large volumes of normal tissues [21]. Here we aimed to extend the analysis of radiotherapy-related changes and radiation dose-effects on the lipid component of serum. MALDI-oa-ToF profiling was applied for the first time to search for radiation-induced changes in human serum lipidome. The positive mode of MALDI ionization was selected in order to favor the analysis of choline-based compounds and other phospholipids, which already have been proposed as potential markers of the response to radiation and anti-cancer treatment [8,10].

## 2. Results

### 2.1. Exposure to Radiation during Radiotherapy Induced Changes in the Serum Lipidome Profiles

**Figure 1.**
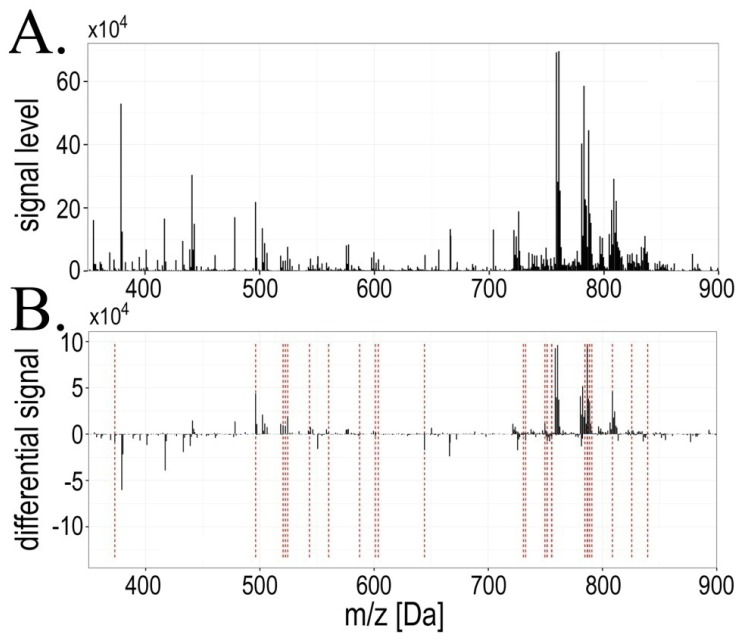
Mass profiles of serum lipids were affected during radiotherapy. (Panel **A**): Averaged mass spectrum of serum lipids registered in the 350–900 Da range for pre-treatment samples (**A**); (Panel **B**): Averaged differential spectrum for pre-treatment and within-treatment samples (AΔB); components that changed their abundances significantly (FDR < 5%) are marked with red lines.

In the analyzed mass range 350–900 Da 842 spectral components (*i.e.*, lipid species with their isotope variants) common for all mass profiles were detected (an average mass profile is presented on Figure 1A). In order to find radiotherapy-related changes individual differential spectra were computed paired with respect to consecutive time points (*i.e.*, changes AΔB, BΔC and AΔC), and then the statistical significance of differences in component’s abundances was estimated (Figure 1B shows resulting differential spectra). Several spectral components changed their abundances significantly between compared time points (FDR < 5% was selected as a statistical significance threshold), which are listed in Table 1 (complete data regarding all registered components are presented in Supplementary Table S1). We observed that major changes occurred between pre-treatment and within-treatment samples (the AΔB change), where 27 spectral components (lipid species) changed their abundance with high level of statistical significance (FDR < 5%). When within-treatment samples were compared with post-treatment samples (the BΔC change), 14 spectral components showed significantly changed abundance. However, abundances of only three spectral components remained different at high level of statistical significance when pre-treatment and post-treatment samples were compared (the AΔC change). Of note, we observed that seven spectral components significantly differentiated samples B from both samples A and samples C (registered *m*/*z* values = 520.36, 522.39, 603.68, 749.51, 760.63, 786.64 and 788.65 Da). Moreover, one spectral component (*m*/*z* value = 751.47 Da) differentiated samples A both from samples B and samples C. Half of the differentiating components were identified with respect to their lipid class (see Table 1), almost all of them being phospholipids containing the choline “head”: phosphatidylcholines (PC; 10 compounds), lysophosphatidylcholines (LPC; 4 compounds) and sphingomyelines (SM; 2 compounds).

### 2.2. Radiotherapy-Related Changes in Lipidome Profiles Showed Different Time-Course Patterns

**Figure 2.**
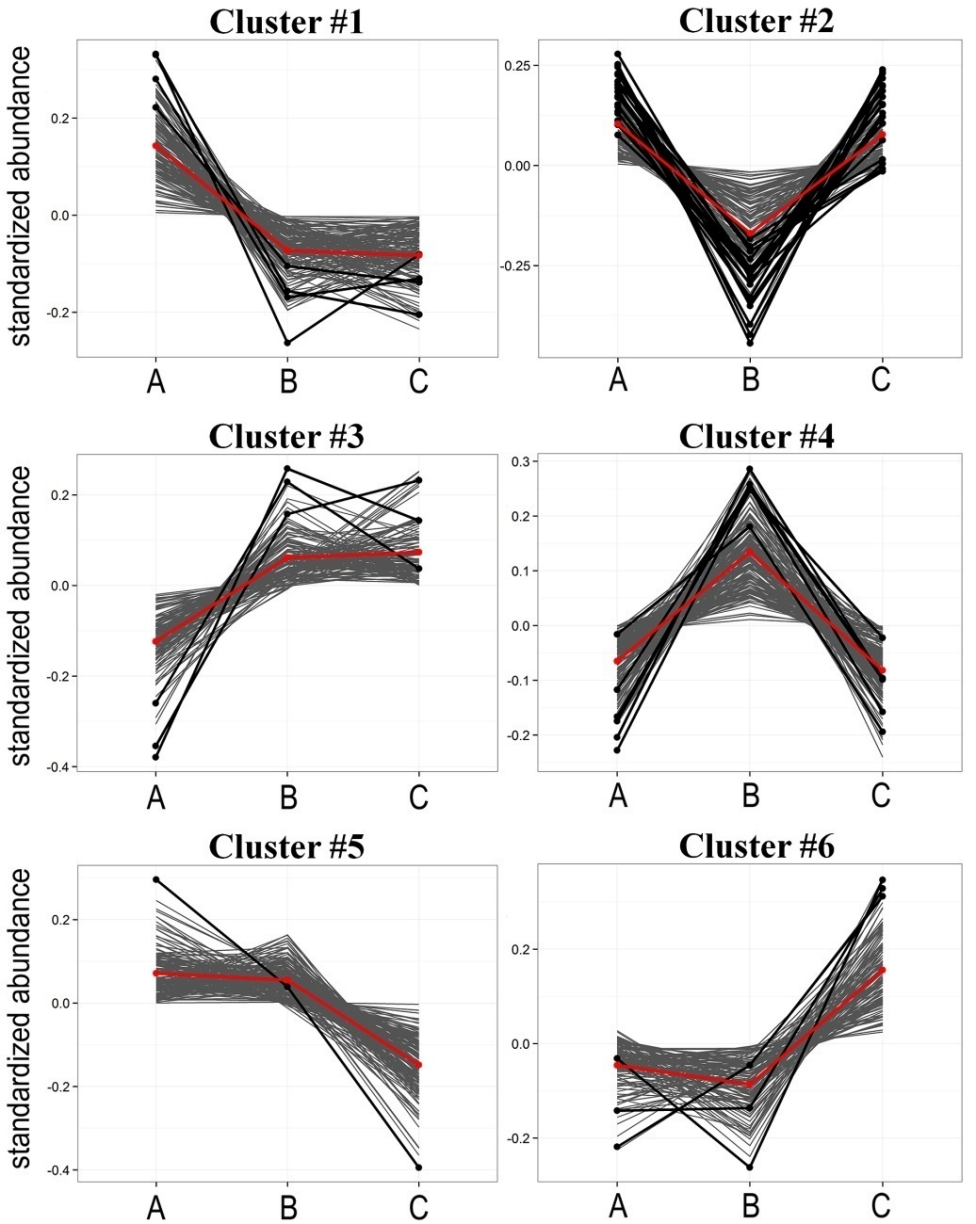
Radiation induced changes followed different patterns. Presented are characteristics of six identified clusters of components with similar time-courses of changes; marked are average profiles for each cluster (red lines) and components that changed abundance significantly (FDR < 5%; solid black lines); other components belonging to each cluster are marked with grey lines.

**Figure 3.**
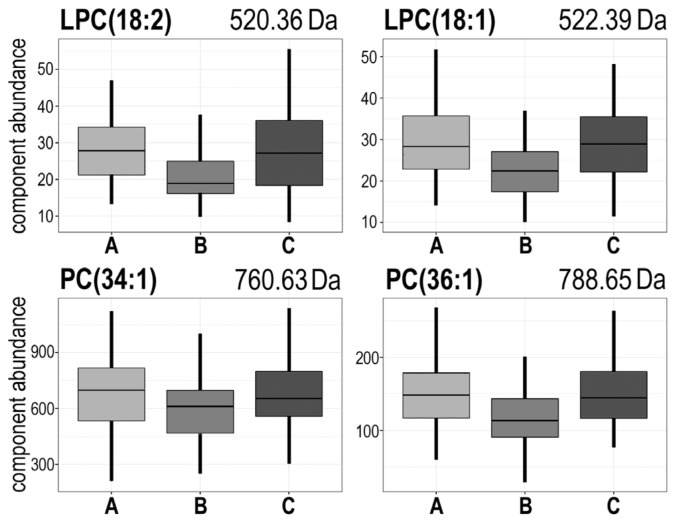
The abundance of several choline-containing phospholipids decreased markedly during radiotherapy and increased afterward. Presented are examples of lysophosphatidylcholines: LPC(18:2) [*m*/z = 520.36 Da] and LPC(18:1) [*m*/*z* = 522.39 Da], and phosphatidylcholines: PC(34:1) [*m*/*z* = 760.63 Da] and PC(36:1) [*m*/*z* = 788.65 Da]. Boxplots show: minimum, lower quartile, median, upper quartile and maximum values (outliers were removed from the plots for perspicuity).

To identify different patterns of radiotherapy-related changes an unsupervised cluster analysis was performed. We identified six different hypothetical patterns of changes (clusters) characterized in Table 2 and depicted in Figure 2 (in case of a few spectral components where differences between time points were not statistically significant observed patterns of changes were not strictly coherent with cluster characteristics); detailed data are presented in Supplementary Table S2. Identified clusters could be further divided into three groups with two “mirrored” clusters in each, where reverse changes were observed: #1 [A > B = C] and #3 [A < B = C], #2 [A > B < C] and #4 [A < B > C], #5 [A = B > C] and #6 [A = B < C]. Of note, the second group (*i.e.*, clusters #2 and #4) where “earlier” changes (AΔB) were compensated by “later” changes (BΔC) was the most numerous (about 70% of all detected components). Furthermore, cluster #2 [A > B < C] contained the majority of differentiating components, that changed abundances significantly between consecutive time points (19 out of 36 “significant” components, see Table 1). This indicated that pattern of changes where “earlier” changes were reversed/compensated by “later” changes was the most common feature of lipidome profiles in serum from irradiated patients. Of note, the majority of “differentiating” LPCs and PCs belonged to cluster #2, and their serum levels decreased significantly during radiotherapy and then increased afterwards; these included LPC(18:2), LPC(18:1), PC(34:1), PC(36:2) and PC(36:1) (Figure 3). On the other hand PCs containing 32 carbons (32:2 and 32:1) and SM(38:3) significantly increased their levels during radiotherapy (cluster #3) (see Table 1).

### 2.3. Radiotherapy-Related Changes in Serum Lipidome Were Associated with Doses of Radiation and Volumes of Irradiated Tissues

**Figure 4.**
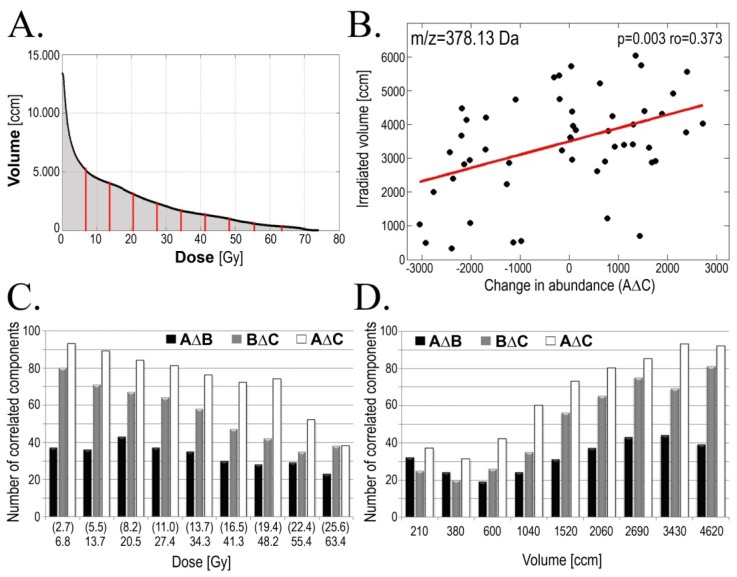
Dose-volume effects in serum lipidome changes. (Panel **A**): Averaged Dose-Volume Histogram; doses corresponding to deciles of the area under curve of the histogram are marked with red lines; (Panel **B**): Correlations between volume of tissue irradiated with 13.7 Gy and changes in abundance of the *m*/*z* = 378.13 Da component in pre- and post-treatment samples (C-A; arbitrary units); Numbers of serum lipidome features correlating with tissue volumes irradiated at a given dose of radiation (Panel **C**) or doses of radiation delivered to a given tissue volume (Panel **D**). Shown here are the AΔB (black bars), BΔC (grey bars) and AΔC (empty bars) changes; *p* = 0.05 was selected as a statistical significance threshold (doses in parentheses refer to AΔB changes).

In the next step of the study we searched for association between features of serum lipidome (*i.e.*, changes in abundance of particular lipidome components) and doses of ionizing radiation received by patients (doses accumulated until a time point corresponding to the collection of sample B in case of the AΔB changes and total doses in case of the AΔC and BΔC changes). Correlations were identified between specific features of the serum lipidome and either the total (maximum) dose received by gross tumor volume (GTV), volume of the patient’s body irradiated at different (smaller) doses or dose delivered to different volume of tissue. Numbers of serum lipidome components, for which changes in abundances correlated with maximum GTV doses are shown in Table 3 (*p* < 0.05 was selected as a statistical significance threshold). We found the highest number of identified correlations was observed in case of AΔC changes (60), yet clear association between maximum GTV doses and features of lipidome were detected also for the AΔB and BΔC changes (44 and 37 components, respectively). The maximum radiation doses (up to 72 Gy) were delivered only to tumor and its adjacent margins (usually 100–200 ccm), while tissue irradiated with lower doses represent much higher volumes (e.g., about 4000 ccm irradiated with 10 Gy). Hence, we searched for correlations between features of serum lipidome and volumes of tissues (including normal tissues irradiated upon IMRT treatment) irradiated with different doses (including “low” or “clinically irrelevant” doses); see Figure 4A,B. Figure 4C shows the numbers of lipidome components, which changes in abundance correlated with volume of tissue irradiated at different doses (*p* = 0.05 was selected as statistical significance threshold). We observed that association between lipidome features and dose-volume effects were the most frequent in case of larger tissue volumes irradiated with clinically low-to-medium doses (*i.e.*, less than 20 Gy in case of the AΔB change and less than 40 Gy in case of the BΔC and AΔC changes; which corresponded to dose fractions below 1 Gy). Additionally, when reverse analysis was performed and serum lipidome features were correlated with doses delivered to a given volume similar results were obtained—majority of correlations were observed for high volumes irradiated with low doses (Figure 4D). Detailed data on correlation of serum lipidome features with volumes irradiated at a given dose or with doses delivered to a given volume are presented in Supplementary Tables S3–S5. Our results clearly indicated that radiotherapy-related changes in serum lipidome profiles depended on doses of delivered radiation, and that low-to-medium doses delivered to large volumes of normal tissue could affect observed changes.

### 2.4. Radiotherapy-Related Changes in Serum Lipidome Were Associated with Radiation Toxicity

Finally, we searched for potential association of serum lipidome features with toxicity of the treatment. The clinically relevant response of normal tissues to toxicity of radiation was assessed using a modified Dische system [22] relying on the intensity of the acute mucosal reaction. The maximum AMR intensity correlates with both maximum GTV dose and volume of normal tissues irradiated with “intermediate” doses (about 0.8–1 Gy per fraction), which was documented in another study based on very similar group of head and neck cancer patients [21]. Here we searched for association between the early radiation toxicity and radiation-induced changes of serum lipidome features. We found correlation between changes in abundance of several lipidome components and the maximum AMR intensity: about 40 lipidome features correlated with the AMR for each of analyzed time-courses (Table 3). Of note, several serum lipidome features associated with the maximum AMR intensity also correlated with volumes of tissues irradiated at given doses of radiation (or radiation doses delivered to a given tissue volume). These features are listed in Table 3. Hence, we concluded that radiotherapy-related changes in the serum lipidome were associated with dose-related toxicity of radiation.

## 3. Discussion

To our knowledge, this is the first paper to analyze the response of human organism to ionizing radiation due to local cancer irradiation performed at the level of blood lipidome. The main changes in abundances of lipid serum components were observed between pre-treatment samples and samples collected during radiotherapy (the AΔB changes). Unsupervised cluster analysis revealed that major group of lipids (70% of registered spectral components) consisted of species, for which radiation-induced changes observed during radiotherapy were reversed/compensated in the post-treatment samples collected 1–2 months after the end of radiotherapy. A minor group of lipids (20% of registered spectral components) consisted of species, where radiation-induced changes detected during radiotherapy remained not reversed/compensated during the follow-up. As a consequence, only a few lipid species showed significant differences when their pre-exposure and post-exposure levels were compared (the AΔC changes). This observation indicated that in case of majority of serum lipids their return to the initial pre-exposure steady-state level was efficient enough during 1–2 months after the end of radiation treatment. Of note, when radiotherapy-related changes in serum proteome profiles were analyzed in a very similar group of patients, the major changes were observed in post-treatment samples collected 1–2 months after the end of radiotherapy (corresponding to the AΔC and BΔC changes). Such serum proteome changes apparently reflected escalation of radiation toxicity (acute mucosal reaction) and its subsequent healing during the follow-up [21]. Here, we show that radiotherapy-related changes in serum lipidome profiles are apparently “faster” compared to changes observed in the low-molecular-weight fraction of serum proteome. In fact, most radiation-induced changes in serum lipidome could be reversed within 1–2 months after completion of radiotherapy, while similar changes in serum proteome could be detected several months after the treatment. This indicated that changes in lipidome and proteome profiles observed in cancer patients treated with radiotherapy might reflect different radiation-induced mechanisms.

Lipid class identification (by MS/MS and/or annotation of registered *m*/*z* values at LipidMaps database) allowed annotation of the majority (85%) of lipids revealing radiation-induced changes as choline-based phospholipids. High extent of lipids referring to this type apparently resulted from both chosen conditions of serum extraction, which favored glycerophospholipids, sphingolipids, sterols and prenols [2], and positive mode of MALDI ionization, which narrowed the ionization of glycerophospholipids to neutral (zwitterionic) representatives, such as phosphatidylocholines and phosphatidylethanolamines [23]. The majority of PLs identified in this work, including different PCs and LPCs as well as SM(34:1), belong to the most abundant in their classes measured in human plasma samples [2]; SM(38:3) and Cer(36:2) are less common species. Phosphatidylocholines are the main building blocks of membrane bilayers and in plasma they are mostly located in high density lipoproteins (HDL). Decreased levels of PCs in serum of irradiated patients may be explained by their rapid turnover in stressed/damaged cells, which resulted in an increased PC’s uptake from the blood. In addition to their main function as a membrane constituent, PCs have a role in signaling through the generation of LPCs (by phospholipase A_2_ enzymes), SMs (by SM synthase), phosphatidic acid (PA; by phospholipase D enzymes) and/or diacylglycerols (DAG; by phospholipase C enzymes). From this point of view, significant down-regulation of major serum PCs observed during radiotherapy might be relevant for increasing capability of cell signaling pathways depending on PC-derived compounds. LPCs are reported to be the major bioactive lipid component of oxidized low density lipoproteins (LDL) and therefore mainly responsible for their pro-inflammatory functions [24]. Down-regulation of LPCs in blood was significantly correlated with activated inflammatory status in many cancer types [25]. Radiotherapy-related down-regulation of LPCs apparently indicated association between inflammatory processes and whole body response to radiation, which was previously documented at the level of serum proteome [21]. Another important class of signaling lipids derived from PCs and LPCs upon action of phospholipase D enzymes are lysophosphatidic acids (LPAs). The most prominent LPA functions include stimulation of cell proliferation, cell survival, and tumor cell invasion [26]. Down regulation of both PCs and LPCs may be therefore explained by the increased formation of LPAs. Another potential mechanism explaining down-regulation of PCs and LPCs involves the disruptive action of reactive oxygen species (ROS) appearing in high levels in irradiated tissues and causing the degradation of these lipids [27]. In contrast to PCs and LPCs, which indicated decreased levels during radiotherapy and were compensated during the follow-up, both identified sphingomyelines showed significant radiation-related up-regulation only: SM(38:3) during earlier stage of the treatment while SM(34:1) during later stage of the treatment or subsequent follow-up. New SM molecules were most probably generated from degraded PC compounds by SM synthase (this transferase utilizes a choline “head” from PC) and suitable ceramide molecules, which was coherent with observed down-regulation of Cer(36:2). SMs can be hydrolyzed back to ceramides by SMase action. This balance between sphingomyeline production and degradation is a key factor in SM-related apoptotic signaling, and generation of ceramides from SMs’ degradation was reported to influence both the rate and form of cell death [28].

Although the model presented here is rather complicated and could be affected by many different processes ongoing in the patient’s organism, one could expect that accumulation and subsequent healing of radiation-induced damage, such as acute mucosal reaction, would have the major influence on general therapy-related changes assessed at the level of serum lipidome. This expectation is supported by observed association of radiotherapy-related serum lipidome features with doses of radiation delivered to normal tissues and intensity of radiation-induced acute mucosal reaction. Although correlations identified here between particular lipidome components and different parameters reflecting radiation doses and toxicity possess moderate statistical power when analyzed separately, reliable conclusions could be drawn based on the general patterns of observed association. Of note, collected data indicated that low-to-medium doses delivered to large volumes of normal tissues during IMRT (considered as “therapeutically irrelevant”) significantly affected whole body response observed at the level of serum lipidome. These observations are consistent with results of our earlier study, where similar association between dose-volume effects and features of the low-molecular-weight fraction of serum proteome has been observed in similar group of head and neck cancer patients [21]. The data indicated collectively, that a whole body response to the local cancer irradiation could be detected at the level of both serum proteome and lipidome. However, the majority of radiation-induced changes in abundances of serum lipids returned to pre-exposure steady-state levels within a relatively short time after the treatment, while changes in serum proteome could be detected even several months after irradiation.

## 4. Experimental Section

### 4.1. Characteristics of the Patients

Sixty-six patients with head and neck squamous cell carcinoma (HNSCC) were enrolled in this study. All of them were Caucasians (64 men) 45–82 years old (median age 63 years); 82% of them were current smokers and 86% alcohol consumers. Cancer was located mainly in larynx (45 pts.), but also in oropharynx (15 pts.) or hypopharynx (6 pts.). The primary tumor stage was scored as: T1 (21%), T2 (44%), T3 (26%) and T4 (9%); 68% of N0. All patients were subjected to IMRT using 6 MeV photons with 1.8 Gy daily fraction doses according to the continuous accelerated irradiation scheme (CAIR) [29]). Total radiation doses delivered to gross tumor volume (GTV) were in the range of 61.2–72 Gy (median 66.6 Gy). Neither surgery nor induction/concomitant chemotherapy was applied to patients enrolled in this study. Three consecutive blood samples (5 mL) were collected from each patient: pre-treatment sample A (within one week before RT; 66 pts.); within-treatment sample B (10–22 days after the start of RT, median 15 days; 66 pts.) and post-treatment sample C (23–59 days after the end of RT, median 36 days; 56 pts.). The acute mucosal reaction (AMR) was estimated using the modified Dische score system [22] every 3–5 days during the radiotherapy. The study was approved by the appropriate Ethics Committee and all participants provided informed consent indicating their conscious and voluntary participation.

### 4.2. Preparation of the Samples

Blood was collected into a 5 mL Vacutainer Tube (Becton Dickinson, Franklin Lakes, NJ, USA), incubated for 30 min RT to allow clotting, and then centrifuged at 1000× *g* for 10 min to remove the clot. The serum was aliquoted and stored at −70 °C until extraction. Total lipids were extracted according to modified Folch method [30]. In brief, 25 μL of serum was mixed with 350 μL of 1:1 methanol/chloroform mixture (*v*/*v*) containing antioxidants: 0.01% (*w*/*v*) 2,6-di-*tert*-butyl-4-methylphenol and 0.005% (*w*/*v*) retinol. The solution was vortex-mixed for 0.5 min and incubated for 30 min at 20 °C. Then 80 μL of water was added to the mixture, vortex-mixed for another 0.5 min and centrifuged (5 min, 10,000× *g*). Chloroform phase (the lower one) was kept and stored at −70 °C until mass spectrometry analysis (within three weeks).

### 4.3. MALDI Mass Spectrometry Analysis

Samples was analyzed using MALDI quadrupole/orthogonal acceleration ToF (oa-ToF) high-definition MS (HDMS) SYNAPT G2-HDMS™ system (Waters, Manchester, UK) equipped with the 355 nm Nd:YAG laser. First, 0.5 μL of sample was mixed directly on stainless steel target plate with 0.5 μL of 30 mg/mL of α-cyano-4-hydroxycinnamic acid (CHCA) matrix (Bruker Daltonics, Billerica, MA, USA) dissolved in 70% (*v*/*v*) acetonitrile containing 0.1% (*w*/*v*) trifluoroacetic acid; each sample was analyzed in triplicate (*i.e.*, placed on three different spots). Mass spectra were recorded using the positive ion mode in the 350–900 Da range with resolution of 10,000 FWHM. Spectra were calibrated with a standard solution of polyethylene glycol (PEG), and *m*/*z* scales were adjusted after acquisition using the PEG signal at *m*/*z* 701.3935 as a lock mass and centroided prior to the generation of accurate mass peak lists. Samples were spotted and analyzed in a random sequence to avoid “batch effect”.

### 4.4. Processing of the Mass Spectra

The initial preprocessing of spectra including alignment, detection of outlier profiles (using Dixon’s Q test), averaging of three technical replicas, additional alignment of averaged individual spectra (*i.e.*, averaged technical replicas), baseline removal and normalization of the total ion current (TIC) was performed according to procedures considering to be standard in the mass spectrometry field [31]. Preprocessed spectra were transferred to Spectrolyzer software suite (v.1.0, MedicWave AB, Halmstad, Sweden; [32]) for peak detection and binning (peak clustering) analysis. The processing steps performed in Spectrolyzer software were also consistent with the standard procedures used for spectral data processing [33,34].

### 4.5. Testing for Differentiating Spectral Components

For each of the 842 spectral components (spectral peaks) statistical significance of differences in abundance between different time points (*i.e.*, samples A, B and C) was estimated using appropriate tools available in R statistical software (see [35]). Individual differential spectra were computed paired with respect to consecutive time points (A–B, B–C and A–C), which resulted in 66 samples for comparison of A *vs.* B, and 56 samples for comparison A *vs.* C and B *vs.* C. To verify whether observed differences in abundances were significant, the Wilcoxon signed rank test was used (with the null hypothesis that the median value of intensities in the differential spectrum is equal to zero). To account for multiple comparisons the Storeys approach [36] that allows for FDR (false discovery rate) control was used. Statistically significant components involved also features that were identified manually as isotopes of other compounds; these components were rejected from the final list of specific components intended for identification.

### 4.6. Identification of Differentiating Components

Spectral components showing significant differences between analyzed time points (FDR < 5%) were analyzed by MS/MS in order to identify lipid class and length of fatty acyl chains. PCs and SM classes were recognized in MS/MS based on characteristic 184.1 Da phosphocholine fragmentation ion, while LPCs based on both 184.1 Da phosphocholine and 104.1 choline fragmentation ions Additionally, other spectral components were annotated at the LipidMaps database [37] based on their registered *m*/*z* values; mass tolerance 0.1 Da and no limit for category/class was applied. Compounds that were not identified experimentally (due to the low abundance of precursor or productions) were regarded as identified only if a single unique lipid record was return from the database search.

### 4.7. Analysis of Patterns of Changes

To investigate the general patterns of changes in abundances of spectral components between compared time points averaged “time courses” were computed based on individual time courses. Data standardization (centering and scaling separately for each of the spectral component) was performed to account for wide differences in abundance ranges observed for distinct components. Cluster analysis was performed using Partitioning Around Medoids (PAM) method, which is a classical algorithm of unsupervised analysis widely used for similar problems [38]. For a given number of clusters (k) the PAM finds k representative objects (so called medoids) that are most different from each other and assigns all the remaining objects to the most similar of the representatives. The similarity of the objects being an input for the PAM was computed based on correlation between average time courses. In order to determine the optimal k number and assess the quality of clustering results, an average Silhouette Width (SW) criterion was used [39], which revealed a six-cluster solution as the optimum.

### 4.8. Correlation of Component’s Abundance with Radiation Parameters

Correlations between changes in abundance of spectral components and parameters reflecting absorbed doses of radiation, as well as maximal intensities of AMR, were analyzed using the Spearman’s rank correlation coefficient. Total radiation dose absorbed by patient’s body was estimated from the individual dose-volume histogram generated during the treatment planning. For the analysis of dose/volume effect we selected the doses corresponding to deciles of the area under the curve of the averaged dose-volume histogram (for details see [21]).

## 5. Conclusions

This study demonstrates for the first time the massive involvement of choline-based lipid serum components in the response of humans to ionizing radiation. Significant change in LPCs’ levels suggests activation of inflammatory processes, while disturbances in levels of sphingomyelines and ceramides indicate involvement of apoptotic pathways. Additionally, correlations of lipidome changes with low and moderate radiation doses call attention to the biological relevance of “therapeutically irrelevant” doses during IMRT.

## Figures and Tables

**Table 1.** Spectral components that changed abundances significantly between analyzed time points. Shown here is the registered *m*/*z* value, significant change in abundance, real pattern of changes, cluster number (hypothetical pattern of changes) and identification (lipid class and length of fatty acyl chains) of analyzed spectral components (*i.e.*, lipid species); components of isotopic envelope were excluded from analysis.

Ion mass[*m*/*z*]	Significant change (FDR < 5%)	Pattern of changes	Cluster number	Lipid class identification
373.08	AΔB	A < B > C	#4	not assigned
496.36	AΔB	A > B < C	#2	**LPC(16:0) + H^+^**
520.36	AΔB;BΔC	A > B < C	#2	**LPC(18:2) + H^+^**
522.39	AΔB;BΔC	A > B < C	#2	**LPC(18:1) + H^+^**
524.38	AΔB	A > B < C	#2	**LPC(18:0) + H^+^**
543.39	AΔB	A > B = C	#2	not assigned
560.28	AΔB	A > B = C	#1	**[Vitamin D3 adduct] + H^+^**
564.64	BΔC	A = B > C	#4	**Cer(36:2) + H^+^**
587.33	AΔB	A < B > C	#4	not assigned
601.12	AΔB	A < B > C	#4	not assigned
603.68	AΔB;BΔC	A < B < C	#6	not assigned
644.11	AΔB	A < B > C	#4	not assigned
703.58	BΔC	A = B < C	#6	**SM(34:1) + H^+^**
721.49	BΔC	A > B < C	#2	not assigned
726.53	AΔC	A = B < C	#6	not assigned
730.62	AΔB	A < B = C	#3	**PC(32:2) + H^+^**
732.47	BΔC	A > B < C	#2	not assigned
732.63	AΔB	A < B = C	#3	**PC(32:1) + H^+^**
749.51	AΔB;BΔC	A > B < C	#2	not assigned
751.47	AΔB;AΔC	A > B = C	#1	not assigned
751.61	AΔB	A < B = C	#4	not assigned
755.42	AΔB	A > B = C	#1	not assigned
755.63	AΔB	A < B = C	#3	**SM(38:3) + H^+^**
758.61	BΔC	A > B < C	#2	**PC(34:2) + H^+^**
760.63	AΔB;BΔC	A > B < C	#2	**PC(34:1) + H^+^**
762.63	BΔC	A > B < C	#2	**PC(34:0) + H^+^**
767.47	BΔC	A > B < C	#2	not assigned
777.33	AΔC	A = B > C	#5	not assigned
784.62	AΔB	A > B < C	#2	**PC(36:3) + H^+^**
786.64	AΔB;BΔC	A > B < C	#2	**PC(36:2) + H^+^**
786.94	AΔB	A > B < C	#2	not assigned
788.65	AΔB;BΔC	A > B < C	#2	**PC(36:1) + H^+^**
790.65	AΔB	A > B < C	#2	**PC(36:0) + H^+^**
808.62	AΔB	A > B < C	#2	**PC(38:5) + H^+^**
825.58	AΔB	A > B = C	#2	not assigned
839.50	AΔB	A > B = C	#4	not assigned

**Table 2.** Characteristics of identified clusters of spectral components.

Cluster	Pattern of change *	Number of components	Number of differentiating components **
#1	A > B = C	147	4
#2	A > B < C	129	19
#3	A < B = C	121	3
#4	A < B > C	170	6
#5	A = B > C	160	1
#6	A = B < C	115	3

* Pattern of change refers to the dominant characteristics of change in the specified cluster (with some not significant deviations from the pattern within the cluster);

** Components which abundances changed significantly between consecutive time points (FDR < 5%).

**Table 3.** Serum lipidome features associated with radiation doses and acute radiation toxicity.

Change	GTV-D	AMR	Examples of components [*m*/*z*] *
AΔB	44	36	473.11; 514.21; 590.61; 872.42
BΔC	37	41	583.61; 669.64
AΔC	60	35	614.38; 641.33; 649.43; 655.65; 673.62; 765.64; 803.71; 886.88

* Components for which abundances correlated with both doses of radiation and radiation toxicity (*p* < 0.05).

## References

[1] Dennis E.A. (2009). Lipidomics joins the omics evolution. Proc. Natl. Acad. Sci. USA.

[2] Quehenberger O., Armando A.M., Brown A.H., Milne S.B., Myers D.S., Merrill A.H., Bandyopadhyay S., Jones K.N., Kelly S., Shaner R.L. (2010). Lipidomics reveals a remarkable diversity of lipids in human plasma. J. Lipid Res.

[3] Marcus A.J., Hajjar D.P. (1993). Vascular transcellular signaling. J. Lipid Res.

[4] Berridge M.J. (1993). Inositol trisphosphate and calcium signaling. Nature.

[5] Wright M.M., Howe A.G., Zaremberg V. (2004). Cell membranes and apoptosis: Role of cardiolipin, phosphatidylcholine, and anticancer lipid analogues. Biochem. Cell Biol.

[6] Bartke N., Hannun Y.A. (2009). Bioactive sphingolipids: Metabolism and function. J. Lipid Res.

[7] Ackerstaff E., Glunde K., Bhujwalla Z.M. (2003). Choline phospholipid metabolism: A target in cancer cells?. J. Cell. Biochem.

[8] Jagannathan N.R., Kumar M., Seenu V., Coshic O., Dwivedi S.N., Julka P.K., Srivastava A., Rath G.K. (2001). Evaluation of total choline from *in vivo* volume localized proton MR spectroscopy and its response to neoadjuvant chemotherapy in locally advanced breast cancer. Br. J. Cancer.

[9] Feurgard C., Bayle D., Guezingar F., Serougne C., Mazur A., Lutton C., Aigueperse J., Gourmelon P., Mathe D. (1998). Effects of ionizing radiation (neutrons/gamma rays) on plasma lipids and lipoproteins in rats. Radiat. Res.

[10] Wang C., Yang J., Nie J. (2009). Plasma phospholipid metabolic profiling and biomarkers of rats following radiation exposure based on liquid chromatography-mass spectrometry technique. Biomed. Chromatogr.

[11] Halperin E.C., Perez C.A., Brady L.W. (2008). Perez and Brady’s Principles and Practice of Radiation Oncology.

[12] Lee N., Puri D.R., Blanco A.I., Chao K.S. (2007). Intensity-modulated radiation therapy in head and neck cancers: An update. Head Neck.

[13] De Neve W., de Gersem W., Madani I. (2012). Rational use of intensity-modulated radiation therapy: The importance of clinical outcome. Semin. Radiat. Oncol.

[14] Rana S., Kumar R., Sultana S., Sharma R.K. (2010). Radiation-induced biomarkers for the detection and assessment of absorbed radiation doses. J. Pharm. Bioallied Sci.

[15] Garaj-Vrhovac V., Kopjar N. (2003). The alkaline comet assay as biomarker in assessment of DNA damage in medical personnel occupationally exposed to ionizing radiation. Mutagenesis.

[16] Kang C.M., Park K.P., Song J.E., Jeoung D.I., Cho C.K., Kim T.H., Bae S., Lee S.J., Lee Y.S. (2003). Possible biomarkers for ionizing radiation exposure in human peripheral blood lymphocytes. Radiat. Res.

[17] Amundson S., Do K., Shahab S., Bittner M., Meltzer P., Trent J., Fornace A.J. (2000). Identification of potential mRNA biomarkers in peripheral blood lymphocytes for human exposure to ionizing radiation. Radiat. Res.

[18] Mah L.J., El-Osta A., Karagiannis T.C. (2010). γH2AX: A sensitive molecular marker of DNA damage and repair. Leukemia.

[19] Menard C., Johann D., Lowenthal M., Muanza T., Sproull M., Ross S., Gulley J., Petricoin E., Coleman C.N., Camphausen K. (2006). Discovering clinical biomarkers of ionizing radiation exposure with serum proteomic analysis. Cancer Res.

[20] Widlak P., Pietrowska M., Wojtkiewicz K., Rutkowski T., Wygoda A., Marczak L., Marczyk M., Polańska J., Walaszczyk A., Domińczyk I. (2011). Radiation-related changes in serum proteome profiles detected by mass spectrometry in blood of patients treated with radiotherapy due to larynx cancer. J. Radiat. Res.

[21] Widlak P., Pietrowska M., Polanska J., Rutkowski T., Jelonek K., Kalinowska-Herok M., Gdowicz-Klosok A., Wygoda A., Tarnawski R., Skladowski K. (2013). Radiotherapy-related changes in serum proteome patterns of head and neck cancer patients; the effect of low and medium doses of radiation delivered to large volumes of normal tissue. J. Transl. Med.

[22] Wygoda A., Maciejewski B., Skladowski K., Hutnik M., Pilecki B., Golen M., Rutkowski T. (2009). Pattern analysis of acute mucosal reactions in patients with head and neck cancer treated with conventional and accelerated irradiation. Int. J. Radiat. Oncol. Biol. Phys.

[23] Fuchs B., Süss R., Schiller J. (2010). An update of MALDI-TOF mass spectrometry in lipid research. Prog. Lipid Res.

[24] Huang Y.H., Schäfer-Elinder L., Wu R., Claesson H.E., Frostegard J. (1999). Lysophosphatidylcholine (LPC) induces proinflammatory cytokines by a platelet-activating factor (PAF) receptor-dependent mechanism. Clin. Exp. Immunol.

[25] Taylor L.A., Arends J., Hodina A.K., Unger C., Massing U. (2007). Plasma lyso-phosphatidylcholine concentration is decreased in cancer patients with weight loss and activated inflammatory status. Lipids Health Dis.

[26] Fang X., Schummer M., Mao M., Yu S., Tabassam F.H., Swaby R., Hasegawa Y., Tanyi J.L., LaPushin R., Eder A. (2002). Lysophosphatidic acid is a bioactive mediator in ovarian cancer. Biochim. Biophys. Acta.

[27] Schiller J., Fuchs B., Arnhold J., Arnold K. (2003). Contribution of reactive oxygen species to cartilage degradation in rheumatic diseases: Molecular pathways, diagnosis and potential therapeutic strategies. Curr. Med. Chem.

[28] Green D.R. (2000). Apoptosis and sphingomyelin hydrolysis: The flip side. J. Cell Biol.

[29] Skladowski K., Maciejewski B., Golen M., Tarnawski R., Slosarek K., Suwinski R., Sygula M., Wygoda A. (2006). Continuous accelerated 7-days-a-week radiotherapy for head-and-neck cancer: Long-term results of phase III clinical trial. Int. J. Radiat. Oncol. Biol. Phys.

[30] Folch J., Lees M., Stanley G.H.S. (1957). A simple method for the isolation and purification of total lipids from animal tissues. J. Biol. Chem.

[31] Hilario M., Kalousis A., Pellegrini C., Müller M. (2006). Processing and classification of protein mass spectra. Mass Spectrom. Rev.

[32] Spectrolyzer Software Suite, MedicWave AB, Halmstad, Sweden (2014). http://www.spectrolyzer.com/spectrolyzer/help-support/manual/.

[33] Cruz-Marcelo A., Guerra R., Vannucci M., Li Y., Lau C., Man T. (2008). Comparison of algorithms for preprocessing of SELDI-TOF mass spectrometry data. Bioinformatics.

[34] Yang C., He Z., Yu W. (2009). Comparison of public peak detection algorithms for MALDI mass spectrometry data analysis. BMC Bioinform.

[35] R Core Team (2014). R: A language and environment for statistical computing.

[36] Storey J.D. (2002). A direct approach to false discovery rates. JRSSB.

[37] Sud M., Fahy E., Cotter D., Brown A., Dennis E.A., Glass C.K., Merrill A.H., Murphy R.C., Raetz C.R.H., Russell D.W. (2006). LMSD: LIPID MAPS structure database. Nucleic Acids Res.

[38] Kaufman L., Rousseeuw P.J. (1990). Finding Groups in Data: An Introduction to Cluster Analysis.

[39] Handl J., Knowles J., Kell D.B. (2005). Computational cluster validation in post-genomic data analysis. Bioinformatics.

